# Extraction of Foreign Bodies from the Bladder

**Published:** 1851-09

**Authors:** 


*Extraction of Foreign Bodies from the Bladder.*—The great difficulty of extracting foreign bodies (not calculi) from the bladder, has led our principal authorities to lay it down as a maxim, "that it is better in all cases to cut into the bladder than to attempt extracting the foreign body by the natural passages." M. Leroy-d'Etiolles, on the other hand, has clearly established that the difficulty depends on the imperfection of the instruments hitherto used for the extraction of such bodies. With proper instruments, such as those employed by M. Leroy-d'Etiolles, a great variety of foreign bodies may be extracted from the bladder through the urethra, and the danger of lithotomy avoided. Since 1841, M. Leroy has performed fourteen operations of this kind, and extracted from different individuals, "the handle of a mustard-spoon, two hair-pins, seven bougies or catheters, two branches of a *brisé-pierre*, and several splinters of bone, which had become neuclei of stones, from man wounded during the revolutions of February and June."

It were impossible to describe the great number of instruments invented by M. Leroy for the above purpose, because they vary with almost every case, according to the nature, size, and form of the obstacle to be removed. It may, however, be mentioned, that M. Leroy, with great liberality, offers to lend them to any surgeon who may have occasion to use them. They will be furnished by M. Mathieu, instrument maker, Rue de l'Ancienne Comedie.—*London Medical Times, from Bul. de l'Acad.*

